# Current status and challenges of robotic surgery in Pakistan

**DOI:** 10.1097/JS9.0000000000000039

**Published:** 2023-03-24

**Authors:** Kaleem Ullah, Sidhant Ochani, Syeda I. Aaqil, Ramsha Haider, Abubakar Nazir

**Affiliations:** a Department of Liver Transplantation, Pir Abdul Qadir Shah Jeelani Institute of Medical Sciences, Gambat; b Department of Medicine, Khairpur Medical College, Khairpur Mir’s; c Department of Medicine, Jinnah Sindh Medical College; d Department of Medicine, Karachi Medical and Dental College, Karachi; e Department of Medicine, King Edward Medical University, Lahore, Pakistan

Robotic surgery is a form of minimal access surgery, also known as robotic-assisted surgery. The Da Vinci Surgical (DVS) system was authorized in 2000. Robotic technology was introduced to facilitate surgeons during challenging operations, keeping the concept of minimal access^1^. Telesurgery is also feasible with the robot, as the surgeon’s console can be connected to a remote network. The first robotic surgery was performed on a patient in Strasbourg, France, by an American physician^2^. Robotic surgery enables surgeons to carry out the surgical procedure with greater accuracy, control, and safety compared to laparoscopic and open approaches^1^. The three-dimensional view and higher magnification in robotic surgery help the surgeon get a depth perception and perform the procedure with greater precision. The quality of nonrestricted moments at the robotic arms compared to laparoscopic surgery makes the procedure effortless. Robotic surgery also eliminates the surgeon’s tremor factor^2^. All these factors help reduce the incidence of visceral injury in patients undergoing robotic surgery. Patients also experience less postoperative pain, decreased hospital stay, and quick return to normal day-to-day activities after robotic procedures^3^. Robotic surgery has been proven to be a very efficient minimally invasive technique, considering its advantages in terms of safety and efficacy. Robotic surgery has a few limitations too. The major hindrance to its availability is its high cost^4^. Also, the majority of the robotic instruments can be used only in a limited number of cases and are discarded thereafter. Moreover, robotic surgery needs dedicated training, which is available only in a limited number of institutes around the world^2^.

In Pakistan, the first DVS system was deployed in 2011 at the Sindh Government Qatar Hospital in Karachi. However, this system collapsed after performing a few procedures, probably due to poor maintenance. The second robotic platform, which was more sophisticated and a generation higher (DVSi) than the previous one, was installed in Civil Hospital Karachi in 2013^5^. More than 500 cases have been performed with this robot in collaboration with the Sindh Institute of Urology and Transplantation (SIUT). The Sindh Government provided funds for only 150 cases under this program^2^. Recently, another robotic system, the cambridge medical robotics Surgical (CMR), a UK-based robotic system, was installed at SIUT Karachi. Over 100 cases have been performed with this system^6^. Moreover, the National Hospital and Medical Centre, a private hospital in Lahore, also reported 13 robotic surgeries^2^.

Robotic surgery in Pakistan is in an infantile phase and is slowly gaining momentum. It is important to review the financial implications and ongoing costs of robotic surgery in Pakistan. The introduction of robotics in public sector hospitals is a quite challenging task in light of Pakistan’s current economic state. The major hurdle and biggest concern in setting up a robotic program in low-income countries like Pakistan is the cost of robotic surgical procedures. Private sectors may be more suitable due to affordability issues, but only a limited number of people can get benefited there^2^. Around 300 million rupees will be needed to install one robotic platform. About 10% of this cost will be needed for annual maintenance and around Rs. 529 310.57 will be needed on a regular basis for the replacement of discarded instruments (roughly after 10 procedures). Also, expenses on robotic surgery training for surgeons will be incurred besides all these^5^.

A substantial investment is needed to install a new robotic platform. Therefore, a comprehensive, well-designed plan focusing on the maintenance, future development, and progress will be needed for the successful adoption of a robotic program in Pakistan, fulfilling international standards. The overall cost, available resources, patient load, suitability, and efficacy of the program should be evaluated before setting it up. To ensure the future success of robotic surgery in Pakistan, the economic model should be designed to be compatible with the country’s health system. For a low-income country like Pakistan, frequent remodeling and tailoring of the program may be essential during the initial phases after regular and vigilant assessments keeping a view on the expenses and available funds for the program, to enhance the efficacy and outcome of the program. The robotic surgery supervision should include a careful financial analysis and market trends. Maintenance, upgradation, recruitment of new team members, and staff training will be needed at regular intervals during various phases of the program. Due to the high installation cost and maintenance expenses, many hospitals may be reluctant to start a robotic program.

For a successful robotic program, a well-trained OR team, devoted and enthusiastic surgeons, supportive hospital administration, sufficient funding sources, and a proper marketing strategy are crucial. For program sustainability, a special focus on marketing should be one of the best strategies, as it can compensate for the high procedure cost by increasing the patient volume.

## Ethical approval

Not applicable.

## Sources of funding

The author(s) received no financial support for the research, authorship, and/or publication of this article.

## Author’s contribution

All authors contributed equally.

## Conflict of interest disclosure

The author(s) declared no potential conflicts of interest concerning the research, authorship, and/or publication of this article.

## Research registration Unique Identifying number (UIN)

Not Applicable.

## Guarantor

Sidhant Ochani.
